# How Consistent Are Consumers in Their Decisions? Investigation of Houseplant Purchasing

**DOI:** 10.3390/bs11050073

**Published:** 2021-05-12

**Authors:** Melinda J. Knuth, Hayk Khachatryan, Charles R. Hall

**Affiliations:** 1 Food and Resource Economics Department, University of Florida, Apopka, FL 32703, USA; Melindaknuth@ufl.edu; 2 Department of Horticultural Sciences, Texas A&M University, College Station, TX 77845, USA; c-hall@tamu.edu

**Keywords:** consumer choice, inconsistency, willingness to pay, price perception, bargain, expensive

## Abstract

This paper examines the impact of intrinsic consumer attributes on decision consistency in houseplant purchasing intentions. Subjects reported their likelihood to buy (LTB) for themselves and as a gift at perceived bargain and getting expensive price levels. The sample was analyzed according to those who switched their LTB ratings to relatively lower values versus subjects who did not by using their demographic characteristics and responses to plant buying behavior questions. Secondly, subjects who had high initial purchase intents were analyzed versus those who had low initial purchase intents. The results indicate that inconsistent purchase decisions are more likely to occur at the perceived getting expensive price level than the perceived bargain price points. Additionally, there are very few demographic differences among the plant buyers who are consistent with their purchase intent versus inconsistent, indicating that external environmental cues may have more of an influence on purchase consistency than intrinsic cues. This information can be utilized by greenhouse and retail firms to understand when a consumer is less likely to change their plant purchase decision with a high initial intent. These price points can help firms optimize their current price offerings within the market and create dialogues with partnering box stores.

## 1. Introduction

Consumer purchasing begins with intent. That is, a purchasing intention initiates the decision-making process a consumer will go through before buying a product. Yet, each purchase intention can be influenced by consumers’ degrees of certainty, and if this certainty outweighs the purchase intention, it can lead to a lost purchase. Previous research regarding plant purchases showed that factors influencing purchase intention include having a guarantee of the viability of the plant, price discounts, and clear, strategic signage [1,2,3]. However, these are all examples of external influences on a purchase decision. What internal influences affect consumer decisions to purchase a plant? Does the intended recipient influence the purchasing decision?

Consumer demand for houseplants has been on the rise since 2015, surging to USD 1.7 billion [4], and insights that could help better predict future demand are applicable to the entire floral supply chain. In a recent survey by the Society of American Florists, 47 percent of consumers indicated an intention to purchase houseplants in the next 6 months, up from 37% from the same survey conducted in 2009, driven by Gen Y and Gen X [5]. Thirty percent of households purchased at least one houseplant last year [6]. However, not all purchase intentions materialize as, increasingly, consumers desire scaled-down, easy-care plants [5]. On average, U.S. households spend $48 on houseplants each year. Millennials have a high appreciation of flowers and really like houseplants (51% purchase intention). They buy on impulse to make themselves feel better. Consumers also indicate that the colors of the flowers and plants help create or express moods and celebrate their personalities [7,8].

As the interest in houseplants is anticipated to continue to rise, it is imperative to evaluate the impact of intrinsic consumer attributes on decision consistency in houseplant purchase intentions, which is the goal of this study. Understanding the extent to which potential consumers make inconsistent purchase decisions can provide important information on how prevalent low purchase certainty is and why it occurs. Additionally, focusing on houseplant consumers who have a high initial certainty to buy and their consistency of their purchase intent can yield beneficial information regarding how to keep their certainty high and what internal aspects (thought processes) of the consumer constitute a change in their high intent. This could provide practical information for industry stakeholders as to how to maintain and increase purchasing consistency for people who have a preexisting high likelihood (intention) to purchase. With this framework in mind, we seek to investigate inconsistent houseplant purchases in consumers’ likelihood to buy (LTB) decisions. Therefore, we have five hypotheses based on the relevant literature:

**Hypothesis** **1(H1).**  *The plant recipient and price level have the same level of saliency in a plant purchase decision.*

**Hypothesis** **2(H2).**  *Plants priced at a discount will have fewer inconsistent LTB decisions than plants priced at regular or high prices.*

**Hypothesis** **3(H3).**  *A person making more inconsistent purchase decisions in their LTB is demographically different than a person with no inconsistent choices.*

**Hypothesis** **4(H4).**  *People who purchase more plants have less inconsistency in their LTB decisions.*

**Hypothesis** **5(H5).**  *Subjects who indicate a high initial likelihood to purchase have a lesser chance of switching because of their buying certainty at the beginning of the decision process.*

## 2. Literature Review

### 2.1. Pricing as a Product Attribute

The price level is generally important to consumers, but its importance relative to other purchasing criteria varies by consumer. Retailers can attract attention and stimulate product sales through communicating discounts and bargains [2]. Grewal et al. [9] showed that price discounts, the individuals’ intrinsic reference values, and their perceptions of the brand quality ultimately influenced the likelihood to purchase through the perceived value of the product. Since there is minimal packaging associated with plants when they are purchased, plant consumers tend to focus on the attributes of the plants themselves [10]. Yet, a little over 15 percent of shoppers are price-oriented, are very sensitive to the price, and fixate on price-related information first, even before viewing the plant. However, during eye-tracking experiments at retail garden centers, some subjects ignored the pricing information altogether [11]. This is thought to be due to the subject’s familiarity with the products, and therefore, they are more reliant on intrinsic cues about the product quality (e.g., the label, container, and signage,) rather than external cues (e.g., price). In conjoint experiments comparing the relative importance of attributes influencing plant purchasing decisions, the price was almost always an attribute of lesser importance [12,13,14,15,16].

### 2.2. United States Houseplant Market

The houseplant category within the plant market was estimated in 2017 to be USD 1.6 billion in value according to the National Gardening Survey [4]. Roughly 30 percent of households have a houseplant within their home. Houseplants are commonly termed to be “entry level” products in the plant market because they can be grown in urban areas and do not require a garden, making them appealing to 18–34-year-olds. Houseplant purchases have increased nearly 5 percent since 2015 [4]. *Nursery Magazine* conducted a survey of independent garden centers where 41 percent of firms saw an increase of 15 percent or more in houseplant sales from 2018 to 2019 [17]. Additionally, 22 percent saw an increase of 5–10 percent, and 11 percent saw an increase in houseplant sales from 11 to 14 percent. Of those purchases, millennials (25–45 years old) are responsible for 31 percent of recent houseplant sales [4]. There is a dearth in formal information reported on houseplant purchasing in 2020 and early 2021.

### 2.3. Plant Purchasing Uses

Within the floral plant category, there is a lack of relevant studies about the factors affecting purchasing decisions for indoor houseplants, since most previous research has focused on cut flowers. However, cut flowers are traditionally thought of and correlated with the market for indoor houseplants (termed foliage plants), as presented by the USDA [18]. The literature has found that consumers, as a consecutive step, begin buying plants for themselves first and transition to buying plants for others as gifts [19,20]. According to recent estimates, 73 percent of consumers buy flowers for themselves at supermarkets due to bargain prices frequently being advertised. Women are more likely to self-purchase cut flowers [21]. Age, marital status, income, fresh floral knowledge, the location grown, and color were statistically significant factors for the self-purchase decision-making process [22].

Local florists are an important venue for gift purchases of cut flowers because of product and service quality perceptions [5,20]. For gift purchasers of flowers, age, being male, having an associate or bachelor’s degree education, income level, direct access, local production, color, and uniqueness all mattered during the selection process [22]. Caplow [23] reported that a primary motivation of gift givers is to fortify important relationships or maintain goodwill with gift recipients. A product perceived as cheap or inexpensive might not fulfill the intended functions they require of the gift. Therefore, a consumer may be willing to pay more for gifts purchased in floral shops because of the additional perceived value (e.g., conspicuous consumption aspects) offered by florists.

Huang [19] compared the decision-making process of floral consumers for both self and gift purchases. Self-purchasers rely more heavily on shop windows and newspapers or magazines as a source of external information than gift purchasers. Gift purchasers also tend to use word-of-mouth recommendations and their personal preferences more in their floral purchasing decisions. Because flower self-purchasers are potentially heavy users of flowers, they may have more floral knowledge than gift purchasers [24,25,26]. Self-purchasers and gift purchasers of flowers behave differently in their external information search, which may result from differences between them in terms of the perceived risk of making incorrect decisions and in terms of expected gains and perceived time, money, energy, or other factors that influence consumer willingness to conduct an external information search [24,25,26]. Gift purchasers focus more on the symbolic meaning and situational value. Self-purchasers focus more on longevity, price discounts, and product quality [24,25,26].

### 2.4. Likelihood to Buy to Evaluate Purchasing Intent

The likelihood to buy scale was adapted from Infosino [27] as a new way to forecast product sales. In essence, this scale was created to further assist with forecasting the Juster scale, which “examines the relationship between purchase intention and purchase behavior for durables” [28]. The question of likelihood to buy (LTB) is based on the concepts of willingness to pay and value [27]. LTB is a common scale used in marketing to assess a consumer’s certainty to purchase [11,13,29,30,31].

When consumers make a purchase, they make this acquisition by participating in four important cognitive steps: recognition of a problem or need, information research, evaluation of the alternatives, and finally, the product choice [32]. After the product choice is made, the process of evaluating how the choice works out begins and influences the consistency that the same choice will be made the next time the desire or need for a similar decision occurs [33]. These choices can be evaluated through using the LTB scale.

### 2.5. Certainty and Consistency in Experiments

Inconsistent decisions have been evaluated in the economic, psychological, and marketing literature via discrete choice experiments. Sælensminde [34] considered an inconsistent “choice” as a decision that is not homogeneous with previous or subsequent decisions, which is congruent with Carlsson et al. [35], defining consistent decisions as making the “same choice in two equal choice tasks”. The reasons for this inconsistency include the complexity of the task at hand [34]; unintended mistakes that do not conform to rational decision-making [36,37,38]; subjects focusing only on one or two attributes [39]; some not caring, leading to inconsistency in answers [40]; and some making a switch intentionally after evaluating their internal switching costs [41]. One theory, passive-bounded rationality, posits that as individuals face increasingly costly choice sets, they continue to attend to all the information in the choice set, but they increasingly make mistakes in processing that information [42,43].

In psychology specifically, there are three main theorems that are used to explain cognitive consistency: Heider’s balance theory, Osgood’s congruity model, and Festinger’s cognitive dissonance theory [44]. Each of these schools of thought seek to address inconsistencies in different ways. Heider’s balance theory postulates that consistency occurs through an interpersonal perspective, while Osgood’s congruity theory “deals with attitude change resulting from the conveyance of information from a source about an object, event or person” [44]. Cognitive dissonance theory is explained as psychological discomforts or a state of disequilibrium amongst one’s cognition (values, beliefs, attitudes, and knowledge) resulting from the inflow of conflicting messages, objects, events, or experiences [44,45]. The cognitive dissonance theory has a wider application, including the use by consumer behavior scientists to understand post-purchase behavior.

In a study evaluating consistency through decision reliability, inconsistency is common in binary options, whether it is between two goods or monetary amounts [46]. Additionally, the response time is longer for early as opposed to later decisions and inconsistent as opposed to consistent decisions, indicating that the response time may be an indicator of decision difficulty [46].

## 3. Materials and Methods

This study was conducted online through the Qualtrics survey platform in early May 2019 and approved through the university’s committee involving research with human subjects (IRB2016-01783). The sample size consisted of U.S. residents of ages 18 years and older. Subjects were recruited using Qualtrics’s recruited subject pool to acquire a random, stratified national sample. All 50 U.S. states were represented through 2094 complete responses (out of a total of 2680 collected respondents).

### 3.1. Study Survey

The survey consisted of (1) screening questions to determine study eligibility, (2) evaluation of 45 miniature houseplant products, (3) a section on houseplant purchases, and (4) a section on respondent sociodemographic characteristics. To participate in the survey, the subject had to pass a series of questions in the screening block to determine if they were interested in purchasing houseplants. The questions related to purchasing houseplants in the past 12 months and how likely they were to purchase houseplants in the future. Exclusion criteria included if the subject did not purchase any plants in the past 12 months. Additionally, subjects were excluded if they failed two quality assurance checks to determine if they were reading the questions. An example of the quality assurance questions is “To ensure that you are reading the question, please select “Yes” as your answer to this statement.” To prevent subject fatigue, each subject randomly rated 11 of the 45 houseplant products.

### 3.2. Houseplant Images and Ratings

Images of houseplants chosen for the study were selected in consultation with industry professionals. The miniature houseplants include the genera of *Anthurium*, *Dracaena*, *Echeveria*, *Ficus*, *Hawthoria*, *Hoya*, *Monstera*, *Mammillaria*, *Juniper*, *Tillandsia*, and *Zanzibar* in sizes ranging from 3 to 15 inches tall. All the houseplants were in or attached to a container including *Tillandsia*. The height of each product was indicated within each image.

In the section of the survey where the houseplants were evaluated, subjects were presented with a product picture and answered three sets of questions. First, subjects were asked to rate their likelihood to buy (LTB) the product for themselves and as a gift for someone else (using a Likert scale, ranging from 0 (very unlikely to buy) to 5 (very likely to buy), which was adapted from Huddleston et al. [11] and Behe et al. [47]). Then, on a follow-up screen, they were asked to indicate at which point the houseplant product price was (1) too low to trust its quality (too cheap), (2) considered to be a bargain price (bargain), (3) getting expensive for their taste, but they would still consider buying it (getting expensive), and (4) too expensive, and they would not consider buying it (too expensive). These four price points were free-response questions, with the requirements that the too cheap price value had to be greater than and not equal to USD 0 and that the higher price level category could not be less than the previous one (for example, the reported too expensive price could not be less than getting expensive or bargain price levels). This method was adapted from the Van Westendorp pricing model [48]. Lastly, the subjects were asked to rate their LTB (using the same Likert scale from 0 (very unlikely to buy) to 5 (very likely to buy) as before) at the bargain and getting expensive price values they input from the previous screen. This three-part process was completed for each of the 11 products the subjects evaluated.

### 3.3. Statistical Analysis

To evaluate our hypotheses, we use two analyses: the overall switching behavior of the subjects and a subsection of subjects who were highly likely to purchase houseplants.

For overall switching behavior, we looked at the consistency of decisions between subjects who were consistent in their decisions versus those who were inconsistent. The LTB ratings for self and gift purchases (initial ratings) were compared to the LTB ratings for the bargain and getting expensive price levels (post ratings), resulting in four treatment groups (Table 1). If the initial ratings were greater in value than the post ratings, an inconsistent decision was noted as a switch in behavior and purchase intent. If the value of the initial rating was less than or equal to the post rating, a consistent decision was noted, and there was no switch in the intent to purchase. Due to the number of inconsistent decisions present, if the subject had at least one switch (out of the 11 miniature houseplant products), they were marked as a switcher (= 1). If there were no switches, they were marked as a non-switcher (= 0).

A follow-up analysis focused only on subjects who rated the houseplant products as likely to buy (rating of 4) or very likely to buy (rating of 5) for themselves or as a gift for someone else. Those who indicated a high initial LTB, and therefore a high probability of purchasing, were denoted as plant buyers. Using the 11 houseplant products, if the subject made an inconsistent decision, they were marked as a non-plant buyer, or if they made consistent decisions, they were marked as a plant buyer (Table 1). To keep consistent with Analysis 1 of the overall sample, if the subject had at least one switch, they were marked as a switcher (= 1), and if they had no switches, they were marked as a non-switcher (= 0). This analysis was done to provide information for horticulture industry stakeholders focusing on consumers who are assumed to be highly interested in plants and plant-related productions.

For both analyses, STATA software (version 16.1, College Station, TX, USA) was used to conduct two sets of four binary logit regressions with the dependent variables of switchers or non-switchers and plant buyers or non-plant buyers. The independent variables were demographic characteristics including gender, income, education, relationship status, region of residency, house type, and population density (metro, suburban, small town, or rural). Additionally, the metrics of the number of plants owned, plant purchasing frequency, and passing the attention check were included.

Two sets of four binary logit regressions were used to evaluate the influence of the reported price levels on the switching behavior for both overall switchers or non-switchers and plant buyer switchers or non-switchers. As before, the dependent variable was each of the four treatment groups (self or gift purchase at the bargain or getting expensive price levels). The independent variables were the aggregate price levels of bargain and getting expensive. The means (or percentage) of each of the demographic variables and price levels are listed in Table 2. A comprehensive list of the levels within each demographic variable is available in Table S1. Additionally, the means and standard deviations of the four price levels are available in Table S2. To compare the number of switchers in the overall sample to the plant buyer switchers, a Pearson’s chi-squared test was used for each of the treatment groups. Compared with the 2019 American Community Survey [49], our survey respondents represented a similar distribution. Census data indicates that the mean household income was USD 65,712. Nationally, 33.1% of Americans had a bachelor’s degree or further education. Females represented 50.8% of the population, and the median age was 38.5 years [49].

### 3.4. Binary Logit Model

Binary logit models were used to analyze the relationship between purchase intention consistency and a set of sociodemographic variables, including gender, income, education level, relationship status, area of residency, house type, purchasing frequency, and number of plants owned. The probability ($P_{i}$) of the *i*th participant’s purchase decision consistency can be represented by

(1) $$P_{i}=\frac{1}{1+{e^{-x^{\prime }}}_{i}}\beta$$

where $x_{i}$ represents participant *i*’s purchasing frequency, the number of plants purchased, and sociodemographic variables and β indicates the estimated coefficients. Marginal effects were then estimated. For continuous variables, the marginal effects indicated the percent change in the probability given a one-unit (or instantaneous change if the unit is very small) increase from the mean in the independent variables. Marginal effects for indicator variables indicated the percent change in the probability when the independent variable increased by one unit or moved from the base attribute level to the level of interest.

## 4. Results

### 4.1. Switchers versus Non-Switchers

**Figure 1:**
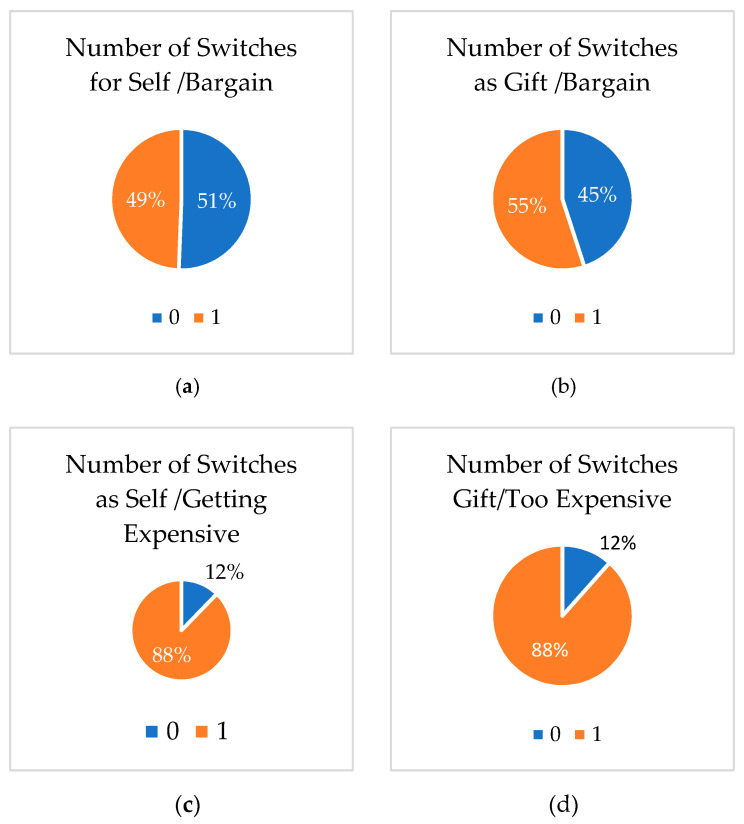
Number of inconsistent decisions of Switchers and Non-Switchers by the treatment group: (**a**) Self Purchase at Bargain price; (**b**) Self Purchase at Getting Expensive price; (**c**) Gift Purchase at Bargain price; (**d**) Gift Purchase at Getting Expensive price.

Half of the respondents (1060 of the 2094) made at least one switch for self-purchases at the bargain price level (Figure 1). At most, two subjects switched all 11 decisions. Nearly all the respondents (1837) made at least one switch for self-purchases at the getting expensive price. Two-thirds of the respondents (1156) made a switch for gift purchases at the bargain price, while nearly all the respondents (1852) made at least one switch for gift purchases at the getting expensive price. There were more switches between the price levels than between the two types of recipients.

Next, we assessed if the individuals who reported a higher price for the bargain and the getting expensive price inputs were more likely to make inconsistent decisions at each price level. Table 3 reflects the marginal effects of the binary logit model for each of the four groups. No matter the reported value, subjects who made a switch at the bargain price level were not less likely to make an inconsistent decision. This was not true for the getting expensive price level. No matter the price reported, subjects at the getting expensive price level for both self and gift purchases were more likely to make a switch at the high price (getting expensive) than the low price (bargain).

Across the four groups, some demographic and plant purchasing behavior consistencies were different between switchers and non-switchers, as indicated by the marginal effects in Table S3. Specific aspects of those differences are discussed below.

Respondents who switched at the self-purchase bargain price were less likely to make a switch if the subject was of a higher age category, compared with the 18–24 age category. If the respondent failed the first attention check, they were 5.8% more likely to make a switching decision for self-purchases at the bargain price. If the person lived in a townhouse, they were 11.9% more likely to make an inconsistent decision than a person who lived in a single-family home, while the remaining housing types had no influence. Lastly, a subject who purchased houseplants monthly was 7.6% more likely to have made a switch in their purchasing decision than a person who bought houseplants weekly.

Males were 5.9% more likely to have made an inconsistent decision than females at the self-purchase, getting expensive price level. If the subject lived in an apartment or a ranch-style home, they were 4.1% or 8.5% more likely, respectively, to make a switch in their purchase intent compared with a subject who lived in a single-family home. If a subject purchased plant yearly for themself at the getting expensive price level, they were 11.4% less likely to make a switch in their purchase intent than a subject who purchased plants weekly.

If the subject failed the attention check, they were 5.8% more likely to make a switch in their purchasing decision at the gift purchase, bargain price level. If the subject made between USD 70,000 and USD 99,999 annually, they were 9.0% less likely to switch compared with a subject who made less than USD 20,000 a year. Subjects who lived in a townhouse were 11.3% more likely to make an inconsistent purchasing decision, and if a subject lived in a dwelling categorized as “other” (did not fit within the other categories of housing), they were 23.1% more likely to make a switch in their purchasing intent.

Very few demographic characteristics had an influence on the switching behavior in the gift purchase, getting expensive treatment category. If a subject purchased plant yearly, they were 12.0% less likely to make a switch in behavior compared with buying weekly. The behavior was not different by age, gender, income, education level, relationship status, population density, region of residency, housing type, the number of plants owned, frequency of purchase, or the attention check results.

### 4.2. Plant Buyer Switchers versus Non-Plant Buyer Switchers

To compare the number of switchers to the number of plant buyers in each treatment group, a Pearson’s chi-squared test was performed to determine if people who purchased more plants had less inconsistency than the overall sample. As confirmed in Table 4 there was no difference in the number of subjects, indicating that plant buyers did not have less inconsistency in their LTB ratings.

**Figure 2:**
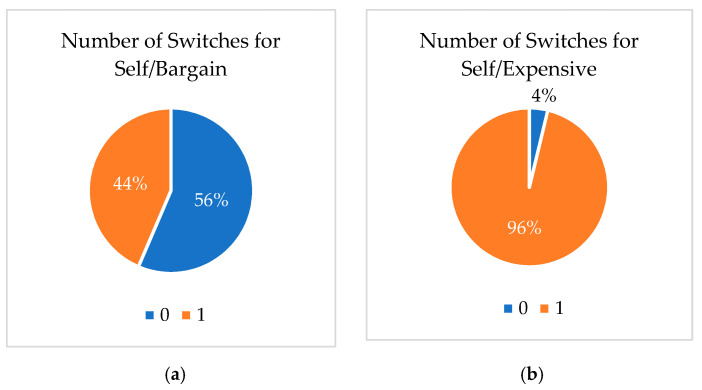

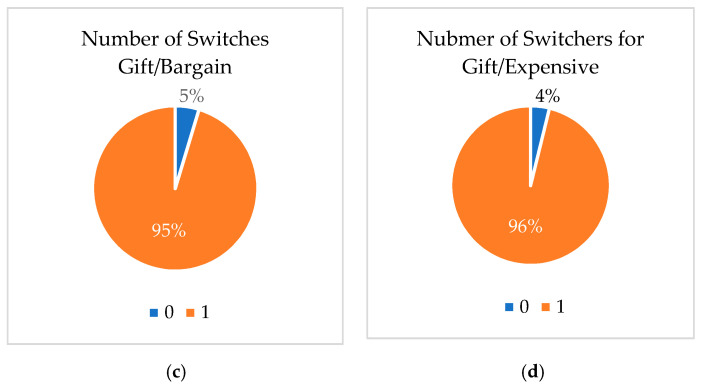
Number of inconsistent decisions of Plant Buyer Switchers and Non-Switchers by the treatment group: (**a**) Self Purchase at Bargain price; (**b**) Self Purchase at Getting Expensive price; (**c**) Gift Purchase at Bargain price; (**d**) Gift Purchase at Getting Expensive price.

Figure 2 provides a visual representation of number of plant buyer switchers and plant buyer non-switchers. Two-thirds of the respondents (1067 of the 1890) made at least one switch for self-purchases at the bargain price level. Nearly all the respondents made a switch for self-purchases at the getting expensive price level as well as for both price levels for gift purchases. Additionally, when looking at the plant buyers by their reported values at both price levels (Table 5), plant buyer switchers at the bargain price level were 1.3% more likely to make a switch at the bargain price. The remaining treatment groups had the same number of switches regardless of their reported prices at either price level. Even though we assumed the plant buyers had strong internal valuations of the product at the beginning of the decision process, they were less consistent with their initial and post LTB ratings.

To assess plant buyers further and potentially determine why they had less certainty than hypothesized, the demographic characteristics were assessed. Table S4 displays the marginal effects of the demographic characteristics of the plant buyers. When looking at the possible internal influences that affect purchases, plant buyers who made inconsistent decisions for purchases for themselves at the bargain price level (self-purchase, bargain) were less likely to make a switch in their purchase intent if they were in the 35–44 (15.6%), 45–54 (21.3%), 55–64 (22.8%), or 65–74 (21.3%) age group. A subject who lived in a townhome was 12.0% more likely to make a switch in their purchase intent than one in a single-family home. Subjects who purchased plants a few times monthly or monthly were 8.6% and 8.7% more likely to make a switch in their purchase intent, respectively, than if purchasing weekly.

For plant buyers who made inconsistent decisions when purchasing for themselves at the getting expensive price level, males were 11.9% more likely to make a switch in their purchase intent than females. If the subject’s income was above USD 70,000 annually, they were 3.5% and 4.0% more likely to make an inconsistent purchasing decision. If a subject was in a relationship but unmarried, they were 3.5% more likely to make a switch in their purchase intent than if they were single. If the subject lived in a townhome or a mobile home, they were 5.0% and 4.7% more likely to make a switch in their purchase intent, respectively, than if living in a single-family home. The more frequent a plant buyer purchased for themselves at the getting expensive price level, the less likely they were to make a switch in purchase intent (few times monthly: 4.8% more likely, monthly: 6.0% more likely, and a few times yearly: 6.7% more likely).

Plant buyers for gift purchases at the bargain price level made less inconsistent decisions if they were 45–54 (5.2% less), 55–64 (3.8% less), or 65–74 (4.9% less) years old compared with 18–24-year-olds. A respondent who owned more than 15 plants was 6.7% more likely to make a switch in their purchase intent compared with a person who had none. A respondent who purchased plants a few times monthly or few times yearly was 2.7% and 4.2% more likely to change their purchase intent, respectively, compared with purchasing weekly.

For plant buying gift purchasers at the getting expensive price level, a respondent in the 45–54 age category was 5.7% less likely to make an inconsistent decision compared with 18–24-year-olds. Additionally, purchasing a few times yearly resulted in a 3.2% higher likelihood of having inconsistent purchase intent compared with purchasing weekly.

## 5. Discussion

### 5.1. Hypothesis 1: Plant Recipient and Price Level Have the Same Level of Saliency in a Plant Purchase Decision

Within the context of this study, it appears that gifting decisions are slightly more inconsistent vis-à-vis self-purchase decisions. Price levels were an important attribute in this experiment. There seemed to be a greater number of decision switches for both self and gift purchases between the two price levels even when they reported their preferred price at each level which indicates the subjects were very price-conscious. This indicates that, unlike in the work of Huddleston et al. [11], the pricing was attended to and greatly considered. The price sensitivity finding is supported by Hovhannisyan and Khachatryan [50], where the price elasticity of foliage plants was found to be more price-responsive than other plant categories and elicited the greatest variability in expenditures. Therefore, H1 (that the plant recipient and price level have the same level of saliency in a plant purchase decision) is not supported, as the price plays a larger part in the decision-making process than the end user of the plant (i.e., self-purchase vs. gift for others). The method in which we presented the LTB and Van Westendorp pricing questions is unique and intellectually involved. The subject’s choice inconsistency was in support of the passive response theorem, where the subjects were trying to track all of the information they were reporting. Yet, the more information they try to be cognizant of, the more errors that they make [42,43]. In the case of this experiment, they tried to track the images of the plants, their LTB ratings for themselves and as a gift, and the four pricing levels, but as the passive response theorem presented, there was an increasing cost to attend to all the information, and therefore, subjects increasingly made mistakes in processing the information. An alternative explanation is that subjects could be relatively neutral about the houseplants and therefore make mistakes because of a lack of attention [40]. This is also supported by the difference the individuals who failed or passed the attention check showed in their inconsistent decisions.

### 5.2. Hypothesis 2: Plants Priced at a Discount will Have Fewer Inconsistent LTB Ratings than Plants Priced at Regular or High Prices

Even though the subjects reported buying plants in the past year, they could be less certain. Therefore, H2 (that plants priced at a discount will have fewer inconsistent decisions than plants priced at regular or high prices) is partially supported.

Though each person’s intrinsic value on a plant was slightly different, the sensitivity of the price was higher at the getting expensive price for purchases, as indicated from the results in Table 3. This theoretical framework is the same for previous works regarding plants and pricing [12,13,14,15,16]. The individuals within this experiment were very price-sensitive. This is perhaps consistent with the findings in Behe et al. [10], where these subjects were price-oriented and determined their purchases based on price alone, and Hovhannisyan and Khachatryan [50], who reported that the demand for ornamental plants is generally price elastic. At the bargain price level, the reported price had no effect on the number of switches, indicating that no matter the price value at this level, it was at the floor of the subject’s monetary threshold, and they would switch depending on other factors.

### 5.3. Hypothesis 3: A Person Making More Inconsistent Decisions in Their LTB Rating is Demographically Different than a Person with No Inconsistent Decisions

Looking at each of the treatment groups for switchers and non-switchers, there are different aspects that influence the consistency of decisions as well, supporting H3 (i.e., a person making more inconsistent decisions is demographically different than a person with no inconsistent decisions). For example, age, gender, and housing type were important characteristics to consider for self-purchasing decision consistency. Yet, there were other demographic characteristics that differentiate the treatment groups from one another, indicating that though the price seemed to be more important in the purchase decision, whom the plant was being purchased for was still considered. Overall, it appears that smaller housing footprints, as in townhouses and apartments, lead to more switching. This may be because of the lack of space available for apartment dwellers or the lack of lighting available through adequate window space.

These differences in consumers seem to derive from the cognitive dissonance theory presented by Festinger [51], where there is a lack of equilibrium in choice consistency based on the inflow of conflicting messages, objects, events, or experiences of the person [44]. In the case of this experiment, the information presented to the subjects did not change through the entire decision-making process, yet there could be conflicting messages or experiences the subject was experiencing based on the subject’s values, beliefs, attitudes, and knowledge that was displaying through their behavior and causing dissonance [52]. Festinger [51] postulated that one of the ways in which cognitive dissonance occurs is through a mistake in a high-involvement decision. Because there were differences between the subjects who made inconsistent decisions versus those who did not, which could be derived from their beliefs, attitudes, and knowledge, causing a higher level of dissonance in their decisions.

At the bargain level for self-purchases, older consumers were more consistent in their purchasing decisions than younger consumers. Additionally, perhaps if a consumer were purchasing more frequently, they would be more likely to accept the asking price if it were below a certain level (as in a bargain) for the product versus a consumer who purchased less frequently. Yet, with the expensive price categories, purchasing yearly led a lesser chance of making a switch in purchase intent. This may be because the consumer had a set yearly price ceiling (a budget) in their mind. As a result, though who were the least-frequent purchasers were willing to commit to the expensive price level, while those who purchased plants the most frequently were more likely to spend their budget on multiple purchasing occasions.

Interestingly, males were more likely to make a switch in purchase intent at the getting expensive level for self-purchases. This finding parallels the cut flower literature (which, historically, houseplants have been grouped with), where females were more likely to make self-purchases and to spend more on their self-purchases than males [19].

### 5.4. Hypothesis 4: People Who Purchase More Plants Have Less Inconsistency in Their LTB Ratings

Another aspect of interest is that if an individual purchase more plants, they are more likely to make a switch in purchase intent. This was false for all four treatment groups. Overall, the number of switchers in the full sample, compared to just plant buyers, was no different. Therefore, H4 (that people who purchase more plants have less inconsistency in their buying certainty) was not supported for plant buyer switchers or plant buyer non-switchers. For plant buyers, the marginal cost for making a switch was relatively small, as indicated by the number of switchers in each of the treatment groups.

### 5.5. Hypothesis 5: Subjects Who Indicate a High Initial Likelihood to Purchase Have a Lesser Chance of Switching Because of Their Buying Certainty at the Beginning of the Decision Process

It is beneficial to know that plant buyers with high likelihoods to purchase initially had the same likelihood to change their decision as a non-plant buyer, even when stating their preferred price. This could indicate that plant buyers do not consider the purchase decision further than an initial impulse response, which is not in support of H5. Additionally, the linear increase in price is not a determinant of decision consistency for plant buyers (Table 5), indicating that other aspects of the purchase decision were at play and the subjects were not as sensitive to price increases at either the bargain or getting expensive price levels. The rejection of H4 and H5 may provide indirect support for the hypotheses that external factors may influence decision consistency in plant purchases. Aspects of the product such as color, signage, viability of the plant, and other retail environmental cues may be “pushing” the product information into the consumer’s decision-making process and, as a result, be the solidifying factor of purchase intent consistency rather than aspects about the consumer themselves [1,2,3,11,38].

Plant buyers were similar in their demographic and behavioral attributes and therefore may be more homogenous in their cognitive processing. As a result, they may also have more similar attitudes, values, and knowledge leading to similar levels of cognitive dissonance [51].

Interestingly, for plant buyers, the price attribute was not as important in the decision-making process as the general consumer. Another way to describe them is that plant buyers are not as sensitive to prices, regardless of if they are bargain or getting expensive prices. Price did not affect the consistence and intent to purchase as much as other potential factors for plant buyers.

As opposed to the overall sample, the less frequently plants were purchased, the more likely there was to be a switch in the purchase decision. This may be, again, because the consumer had a set yearly price ceiling (a budget) in their mind. It could also be that more frequent plant buyers gain more positive utility from purchasing than plant buyers who buy less frequently, especially for self-purchases. 

## 6. Conclusions

The objective of this study was to evaluate the impact of intrinsic consumer attributes on decision consistency in plant purchasing intentions. Specifically, it quantified the extent to which houseplant purchasers who may have started with a high intent to purchase later lost certainty in their likelihood to buy, leading to inconsistent decision behaviors. Retail industry stakeholders frequently rely on stated preferences when transactional data are not accessible (e.g., new product introduction) to adjust or optimize pricing and other relevant retail marketing practices. The results in this study showed that hypothetical commitment bias partially exists, even when the price points are suggested by the consumer. Given consumers’ high sensitivity to changes in ornamental plant prices, it is especially important to understand the circumstances under which potential customers are likely to switch. For frequent houseplant buyers, inconsistent purchasing intention behavior is just as likely as in the public. However, very little distinguishes what causes a plant buyer to make an inconsistent decision. The passive reaction theorem would be less likely to hold for plant buyers, because all four of the plant buyer treatment groups were not different in their decision consistency when considering the attention check failure. This suggests that whether they passed the attention check or not, the subjects had similar decision consistencies. It could be that they, again, were considering all the information and simply made a mistake. Alternatively, it could be that external, retail environmental cues have a stronger influence on the consistency of purchase intent and play a greater role than internal cues.

### 6.1. Managerial Implications

This information can be utilized by firms to understand when a consumer is less likely to change their decision to purchase after arriving at the retail firm or website with a high initial intent to purchase a plant. Additionally, consumers are more likely to change their decisions if the price is higher, even if they set the price values themselves. The results also revealed that inconsistent purchasing behaviors (e.g., switching from an initial high intent to purchase to lesser or no intent) exist for both self and gift purchase intentions.

The older the plant buyer, the less likely they were to make a switch in their purchase intent at the bargain price level for self and gift purchases. This was also true for the overall sample for self-purchases. Older consumers may be more established plant purchasers than younger consumers. They have been purchasing houseplant products longer and therefore may be more confident in their decisions to purchase than younger consumers, who may be just entering the market with a discretionary income or establishing themselves as plant purchasers. Older consumers were also less likely to change their decisions to purchase plants for themselves or as gift purchases. Additionally, if a consumer purchases plants less frequently, they are more likely to be inconsistent with their purchase intent.

These price points can help greenhouse and retail firms optimize their current price offerings within the market. Greenhouse firms can bring the likelihood to purchase and inconsistent decision information into dialogues with partnering box stores to assist with negotiations, specifically bringing exclusive houseplant-related programs so that box stores are able to differentiate the houseplant category within the home improvement marketplace.

It is important to understand what internal cues can change consumers’ purchase decisions and, ultimately, prevent them from buying. However, providing external cues such as signage, plant tags, displays, and adequate information about the plant are important aspects that the firm can employ to assist the consumer in their purchasing decision. Future work can include questions about the subjects’ knowledge related to plant care, how much they enjoy purchasing plants, and if their knowledge, mood, and enjoyment play a role in purchase intent consistency.

### 6.2. Limitations and Future Research

Our current findings focused on one product category, which may not translate to other plant products or other products in general. Therefore, future work can focus on other plant-related categories using the same methodology. Additionally, we examined internal cues that affect decision consistency but ignored external cues. Future research looking at decision consistency with different environmental cues may create a more holistic view of consumer decision consistency.

## Figures and Tables

**Table 1:** Four treatment groups used to analyze inconsistency in likelihood to buy.

	Analysis 1	
	Switchers	Non-Switchers
	Follow-up LTB < Initial LTB	Follow-up LTB ≥ Initial LTB
	Purchase by Recipient (Self vs. Gift for Others)	
**Price Level**	Self-Purchase, Bargain Price	Gift Purchase, Bargain Price
	Self-Purchase, Getting Expensive Price	Gift Purchase, Getting Expensive Price
	**Analysis 2**	
	Non-Plant Buyers	Plant Buyers
	Follow-up LTB < Initial LTB of 4	Follow-up LTB ≥ Initial LTB of 4
	Purchase by Recipient (Self vs. Gift for Others)	
**Price Level**	Self-Purchase, Bargain Price	Gift Purchase, Bargain Price
	Self-Purchase, Getting Expensive Price	Gift Purchase, Getting Expensive Price

**Table 2:** Demographic characteristics (independent variables) of the sample.

Demographic Characteristic	Definition	Mean or %	SD
Age	Years (no.)	38.9	13.5
Gender	0 = male; 1 = female	0.56	0.50
Income	Average of levels	USD 58,923.04	USD 44,692.92
	USD < 20–39.9 k	42.38%	
	USD 40–69.9 k	28.54%	
	USD 70–99.9 k	14.37%	
	USD > 100 k	14.70%	
Education	Some high school or less	1.95%	
	High school diploma or GED	20.85%	
	Some college courses	26.08%	
	Associate degree	13.61%	
	Bachelor’s degree	23.49%	
	Some graduate school	2.18%	
	Graduate or professional’s degree	11.83%	
Relationship Status	Not married or single	23.72%	
	In a relationship	12.29%	
	Married	45.15%	
	Divorced or separated	14.42%	
	Widowed	4.42%	
Region of Residency	Northeast	4.42%	
	Mid-Atlantic	15.80%	
	Midwest	21.83%	
	South	29.64%	
	Southwest	11.77%	
	West	16.54%	
House Type	Single-family home	60.60%	
	Townhouse	4.71%	
	Condominium	3.22%	
	Multi-family home	3.39%	
	Apartment	18.84%	
	Co-op	0.289%	
	Ranch style home	2.58%	
	Mobile home	5.40%	
Population Density	Major town or city	24.12%	
	Suburban	43.08%	
	Small town	13.04%	
	Rural area	19.76%	
Number of Plants Owned	None	6.15%	
	1	11.95%	
	2–5	51.87%	
	6–10	20.85%	
	11–15	5.17%	
	Over 15	4.02%	
Plant Purchasing Frequency	Once a week or more	22.57%	
	2–3 times monthly	43.88%	
	Once monthly	16.83%	
	2–3 times yearly	12.35%	
	Once yearly	12.35%	
	Do not purchase at all	4.37%	
Attention Check	0 = passed; 1 = failed	0.30	0.47

**Table 3:** Marginal effect estimates from four binary logit models summarizing the effects of the reported bargain and getting expensive prices on switching behavior between switchers and non-switchers (*n* = 2094).

Switchers and Non-Switchers								
	Self-Purchase, Bargain		Self-Purchase, Getting Expensive		Gift Purchase, Bargain		Gift Purchase, Getting Expensive	
**Variables ^z^**	**dy/dx ^y^**	**SE**	**dy/dx**	**SE**	**dy/dx**	**SE**	**dy/dx**	**SE**
Bargain Price	0.0035	<0.01	**−0.0032**	<0.01	**0.0027**	<0.01	**−0.0030**	<0.01
Getting Expensive	0.0002	<0.01	**0.0006**	<0.01	0.0001	<0.01	**0.0005**	<0.01
Log Likelihood	−1444.9869		−774.0785		−1436.0519		−744.1785	
LR χ^2^	12.60		11.17		8.06		10.95	
Prob > χ^2^	0.0018		0.0038		0.0178		0.0042	
Pseudo R^2^	0.0043		0.0072		0.0028		0.0073	

^z^ Bold font indicates significance at *p*-values ≤ 0.05. ^y^ Marginal effects.

**Table 4:** Comparison of the proportion of subjects as switchers and plant buyers in each of the four treatment groups.

Treatment Groups Switchers (*n* = 2094) vs. Plant Buyers (*n* = 1890)	Chi-Squared, *p*-Value
Self Purchase at the Bargain Price	0.3584, 0.5490
Self Purchase at the Getting Expensive Price	0.6341, 0.4260
Gift Purchase at the Bargain Price	1.5683, 0.2100
Gift Purchase at the Getting Expensive Price	0.2957, 0.5870

**Table 5:** Marginal effect estimates from four binary logit models summarizing the effects of the reported bargain and getting expensive prices on the switching behavior between plant buyer switchers and plant buyer non-switchers (*n* = 1813).

Plant Buyer Switchers and Plant Buyer Non-Switchers								
	Self-Purchase, Bargain		Self-Purchase, Getting Expensive		Gift Purchase, Bargain		Gift Purchase, Getting Expensive	
Variables ^v^	dy/dx ^u^	SE	dy/dx	SE	dy/dx	SE	dy/dx	SE
Bargain Price	**0.0125**	0.01	0.0001	<0.01	−0.0003	<0.01	−0.0003	<0.01
Getting Expensive	−0.0016	<0.01	0.0017	<0.01	0.0017	<0.01	0.0017	<0.01
Log Likelihood	−1224.9215		−292.2543		−346.2265		−296.3763	
LR χ^2^	26.38		8.35		5.71		6.52	
Prob > χ^2^	0.0000		0.0154		0.0575		0.0384	
Pseudo R^2^	0.0107		0.0141		0.0082		0.0109	

^v^ Bold font indicates significance at *p*-values ≤ 0.05. ^u^ Marginal effects.

## Data Availability

Not applicable.

## Supplementary Materials

The following are available online at https://www.mdpi.com/article/10.3390/bs11050073/s1, Table S1: Levels of each demographic characteristics, Table S2: The price levels of the sample, Table S3: Marginal effects estimate from four binary logit models summarizing the effects of sociodemographic characteristics, attention check, and reported houseplant purchasing habits in-fluence on switching behavior between Switchers and Non-Switchers (*n* = 2094), Table S4: Summary of sociodemographic characteristics, attention check, and reported prices in-fluence on switching behavior for Plant Buyer treatment groups.
