# Supplementary material for: How Consistent Are Consumers in Their Decisions? Investigation of Houseplant Purchasing
Source: Behav Sci (Basel). 2021 May 12;11(5):73. doi: 10.3390/bs11050073 (PMC8151653; doi:10.3390/bs11050073)
Supplement: Supplementary file 1 [file behavsci-11-00073-s001.zip › behavsci-1179730-supplementary.pdf]

Supplementary Materials. Tables

Table S1. Levels of each demographic characteristics

Demographic Characteristic	Level	Value
Education	Some high school or less	1
	High school diploma/GED	2
	Some college courses	3
	Associate degree	4
	Bachelor's degree	5
	Some graduate school	6
	Graduate or professional's degree	7
Relationship Status	Not married/single	1
	In a relationship	2
	Married	3
	Divorced/separated	4
	Widowed	5
Annual Income	Less than $20,000	1
	$20,000 - $29,999	2
	$30,000 - $39,999	3
	$40,000 - $49,999	4
	$50,000 - $59,999	5
	$60,000 - $69,999	6
	$70,000 - $79,999	7
	$80,000 - $89,999	8
	$90,000 - $99,999	9
	$100,000 - $149,999	10
	$150,000 - $199,999	11
	Over $200,000	12
House Type	Single-Family Home	1
	Townhouse	2
	Condominium	3
	Multi-Family Home	4
	Apartment	5
	Co-op	6
	Ranch Style Home	7
	Mobile Home	8
Area of Residency	Major Town/City	1
	Suburban	2
	Small Town	3
	Rural Area	4
Region	Northeast	1
	Mid-Atlantic	2
	Midwest	3
	South	4
	Southwest	5
	West	6
Number of Houseplants	None	1
	1	2
	2-5	3
	6-10	4
	11-15	5
	Over 15	6
Plant Purchase Frequency	Once a week or more	1
	2-3 times monthly	2
	Once monthly	3
	2-3 times yearly	4
	Once yearly	5
	Do not purchase at all	6

Table S2. The price levels of the sample.

Price Level (US Dollar)	Mean	SD
Too Cheap Price Level	$6.98	$5.60
Bargain Price Level	$10.85	$6.53
Getting Expensive Price Level	$17.47	$10.65
Too Expensive Price Level	$25.90	$16.99

Table S3. Marginal effects estimates from four binary logit models summarizing the effects of sociodemographic characteristics, attention check, and reported houseplant purchasing habits influence on switching behavior between Switchers and Non-Switchers (n=2094).[z]

		Switchers and Non-Switchers							
		Self Purchase/ Bargain		Self Purchase/ Getting Expensive		Gift Purchase/ Bargain		Gift Purchase/ Getting Expensive	
Variables[x]		dy/dx[w]	SE	dy/dx	SE	dy/dx	SE	dy/dx	SE
Age	18-24	(base)							
	25-34	-0.07060	0.05	-0.0170	0.03	0.0200	0.05	-0.0216	0.03
	35-44	**-0.09976**	0.05	-0.0183	0.03	-0.0509	0.05	-0.0188	0.03
	45-54	**-0.15113**	0.05	-0.0459	0.03	-0.1024	0.05	-0.0318	0.03
	55-64	**-0.16458**	0.05	-0.0342	0.03	-0.0788	0.05	-0.0124	0.03
	65-74	**-0.14348**	0.05	-0.0559	0.03	-0.0869	0.05	-0.0449	0.03
Gender	1= Female	0.03103	0.02	**0.0590**	0.02	-0.0359	0.02	0.0160	0.01
Attention Check	1=failed	**0.05793**	0.02	-0.0251	0.02	**0.0575**	0.02	-0.0254	0.02
Income	< $20k-39.9	(base)							
	$40k-69.9	0.02260	0.03	-0.0305	0.02	-0.0231	0.03	-0.0092	0.02
	$70k-99.9	-0.04281	0.04	-0.0222	0.02	**-0.0903**	0.04	-0.0302	0.02
	> $100k	-0.03881	0.04	-0.0325	0.02	-0.0574	0.04	-0.0183	0.02
Education	Some high school	(base)							
	High school	-0.00075	0.07	0.0416	0.06	0.0253	0.07	0.0059	0.05
	Some college	-0.00020	0.07	0.0861	0.06	0.0691	0.07	0.0483	0.05
	Associate	0.01389	0.08	0.1022	0.06	0.0829	0.08	0.0334	0.05
	Bachelor	-0.01145	0.08	0.1124	0.06	0.0741	0.08	0.0652	0.05
	Some graduate school	0.11857	0.10	0.1240	0.07	0.1283	0.10	0.0856	0.06

	MS, PHD, Profes-sional De-gree	0.00298	0.08	0.0834	0.06	0.0839	0.08	0.0016	0.06
Relationship Status	Single	(base)							
	In relation-ship	-0.02927	0.04	0.0145	0.02	0.0715	0.04	0.0397	0.02
	Married	-0.02132	0.03	-0.0095	0.02	0.0228	0.03	0.0208	0.02
	Divorced	0.02325	0.04	0.0313	0.02	0.0246	0.04	-0.0005	0.03
	Widowed	-0.06123	0.06	-0.0499	0.05	-0.0256	0.06	-0.0420	0.05
Population Density	Metro	(base)							
	Suburban	0.00757	0.03	-0.0025	0.02	0.0164	0.03	0.0044	0.02
	Small Town	-0.03814	0.04	0.0276	0.02	-0.0140	0.04	0.0433	0.02
	Rural Area	0.02596	0.04	0.0048	0.02	0.0567	0.04	0.0150	0.02
Region	Northeast	(base)							
	Mid-Atlan-tic	-0.07053	0.06	-0.0353	0.04	-0.0004	0.06	-0.0036	0.04
	South	-0.04431	0.06	-0.0147	0.04	0.0017	0.06	-0.0105	0.04
	Midwest	-0.04147	0.06	-0.0234	0.04	0.0165	0.06	0.0086	0.04
	Southwest	0.00221	0.06	-0.0341	0.04	0.0844	0.06	0.0160	0.04
	West	-0.00427	0.06	0.0138	0.04	0.0441	0.06	0.0226	0.04
House Type	Home	(base)							
	Town-house	**0.11921**	0.05	0.0060	0.04	**0.1127**	0.05	0.0378	0.03
	Condo	0.08721	0.06	0.0042	0.04	0.0362	0.06	0.0133	0.04
	Multi-Fam-ily Home	0.08593	0.06	0.0079	0.04	0.0404	0.06	0.0179	0.04
	Apartment	0.04565	0.03	**0.0411**	0.02	-0.0293	0.03	0.0088	0.02
	Co-op	0.04212	0.19	0.0141	0.12	-0.0086	0.19	-0.1392	0.16
	Ranch Style Home	0.01711	0.07	**0.0850**	0.03	-0.0492	0.07	0.0104	0.05
	Mobile Home	0.08673	0.05	0.0448	0.03	0.0131	0.05	0.0153	0.03
	Other	0.03361	0.11	-0.0241	0.08	**0.2309**	0.09	0.0667	0.05
Number of Plants Owned	None	(base)							

	1 plant	0.07599	0.05	0.0323	0.04	0.0657	0.05	0.0329	0.03
	2-5 plants	0.05669	0.05	0.0219	0.03	0.0779	0.05	0.0283	0.03
	6-10 plants	0.05221	0.05	0.0159	0.04	0.0773	0.05	0.0181	0.04
	11-15 plants	0.00940	0.07	-0.0438	0.05	0.0029	0.07	-0.0170	0.05
	More than 15 plants	-0.00144	0.07	-0.0069	0.05	-0.0676	0.07	-0.0151	0.05
Plant Purchase Frequency	Weekly	(base)							
	Few Times Monthly	0.04475	0.03	0.0146	0.02	-0.0083	0.03	0.0126	0.02
	Monthly	**0.07635**	0.04	0.0010	0.02	0.0172	0.04	-0.0052	0.02
	Few Times Yearly	0.05612	0.04	-0.0387	0.03	-0.0375	0.04	-0.0408	0.03
	Yearly	-0.00486	0.06	**-0.1137**	0.05	-0.0934	0.06	**-0.1196**	0.05
Log Likelihood		-1413.5053		-728.4804		-1401.3364		-714.9900	
LR χ^2		75.57		102.37		77.49		69.33	
Prob> χ^2		0.0029		0.0000		0.0019		0.0114	
Pseudo R^2		0.0260		0.0656		0.0269		0.0462	

[x]Bold font indicates significance at P-values ≤ 0.05.
[w]Marginal effects.

Table S4. Summary of sociodemographic characteristics, attention check, and reported prices influence on switching behavior for Plant Buyer treatment groups.

		**Plant Buyer Switchers and Plant Buyer Non-Switchers**							
		**Self-Purchase/ Bargain**		**Self-Purchase/ Getting Expensive**		**Gift Purchase/ Bargain**		**Gift Purchase/ Getting Expensive**	
**Variables[t]**		**dy/dx[s]**	**SE**	**dy/dx**	**SE**	**dy/dx**	**SE**	**dy/dx**	**SE**
Age	18-24	(base)							
	25-34	-0.10696	0.05	-0.0280	0.02	-0.0256	0.02	-0.0114	0.01
	**35-44**	**-0.15643**	**0.05**	-0.0068	0.02	-0.0138	0.01	-0.0067	0.01
	**45-54**	**-0.21276**	**0.05**	-0.0164	0.02	**-0.0520**	**0.02**	**-0.0509**	**0.02**
	**55-64**	**-0.22770**	**0.05**	-0.0078	0.02	**-0.0384**	**0.02**	-0.0284	0.02
	**65-74**	**-0.21248**	**0.05**	-0.0501	0.02	**-0.0492**	**0.02**	-0.0332	0.02
Gender	1 = Female	0.00343	0.02	**0.0119**	**0.01**	-0.0163	0.01	-0.0170	0.01
Attention Check	1 = failed	0.03972	0.02	-0.0043	0.01	-0.0081	0.01	-0.0039	0.01
Income	< $20k-39.9	(base)							
	$40k-69.9	0.03422	0.03	0.0208	0.01	-0.0067	0.01	0.0066	0.01
	$70k-99.9	-0.02946	0.04	**0.0345**	**0.02**	0.0074	0.02	0.0079	0.01
	>$100k	-0.03653	0.04	**0.0399**	**0.02**	-0.0079	0.02	-0.0104	0.02

Education Level	Some high school	(base)							
	High school	-0.00794	0.08	-0.0214	0.03	0.0967	0.07	0.0283	0.05
	Some college	-0.02987	0.08	-0.0115	0.03	0.0969	0.07	0.0272	0.05
	Associate	-0.04386	0.08	-0.0346	0.03	0.1030	0.07	0.0287	0.05
	Bachelor	-0.05232	0.08	-0.0262	0.03	0.1029	0.07	0.0379	0.05
	Some graduate school	0.03032	0.11	-0.0287	0.05	0.1179	0.07	.	.
	MS, PHD, Professional Degree	-0.01398	0.09	-0.0372	0.03	0.0780	0.07	0.0247	0.05
Relationship Status	Single	(base)							
	In relationship	0.02004	0.04	**0.0352**	**0.02**	0.0158	0.02	0.0100	0.02
	Married	0.00530	0.03	0.0193	0.02	0.0245	0.02	0.0108	0.02
	Divorced	-0.01810	0.04	0.0038	0.02	0.0221	0.02	0.0236	0.02
	Widowed	-0.05329	0.06	0.0295	0.02	0.0306	0.02	0.0247	0.02
Population Density	Metro	(base)							
	Suburban	0.03601	0.03	0.0093	0.01	0.0040	0.01	0.0066	0.01
	Small Town	-0.03633	0.04	-0.0034	0.02	0.0210	0.02	0.0089	0.02
	Rural Area	0.03055	0.04	-0.0042	0.02	0.0164	0.02	0.0128	0.02
Region	Northeast	(base)							
	Mid-Atlantic	-0.03655	0.06	-0.0328	0.03	0.0041	0.03	0.0131	0.03
	South	-0.03569	0.06	-0.0147	0.02	0.0070	0.03	0.0148	0.03
	Midwest	-0.01655	0.06	-0.0211	0.02	0.0143	0.03	0.0260	0.03
	Southwest	0.04092	0.06	-0.0090	0.03	0.0132	0.03	0.0258	0.03
	West	-0.02573	0.06	-0.0210	0.03	-0.0141	0.03	-0.0125	0.03
House Type	Home	(base)							
	Townhouse	**0.12018**	**0.05**	**0.0496**	**0.01**	0.0179	0.02	0.0121	0.02
	Condo	0.07853	0.07	0.0052	0.03	0.0021	0.02	-0.0082	0.03
	Multi-Family Home	0.07418	0.06	.	.	-0.0558	0.04	-0.0271	0.04
	Apartment	0.02395	0.03	0.0173	0.01	-0.0093	0.01	-0.0042	0.01
	Co-op	0.19699	0.20	.	.	-0.2428	0.21	-0.2622	0.22
	Ranch Style Home	0.00610	0.07	-0.0022	0.04	-0.0799	0.05	-0.0859	0.05
	Mobile Home	0.07985	0.05	**0.0467**	**0.01**	-0.0176	0.03	-0.0158	0.02

	Other	-0.08569	0.12	.	.	-0.0425	0.06	-0.0411	0.05
Number of Plants Owned	None	(base)							
	1 plant	0.03787	0.05	0.0196	0.02	0.0297	0.03	0.0392	0.03
	2-5 plants	-0.00705	0.05	0.0131	0.02	0.0338	0.03	0.0464	0.03
	6-10 plants	0.02856	0.05	-0.0037	0.03	0.0297	0.03	0.0424	0.03
	11-15 plants	0.02569	0.07	0.0470	0.03	0.0129	0.04	0.0425	0.03
	More than 15 plants	0.00974	0.07	0.0295	0.03	**0.0670**	0.03	0.0703	0.03
Plant Purchase Frequency	Weekly	(base)							
	Few Times Monthly	0.05823	0.03	**0.0483**	0.02	**0.0271**	0.02	0.0175	0.01
	Monthly	**0.08649**	**0.04**	**0.0595**	0.02	0.0302	0.02	0.0201	0.02
	Few Times Yearly	**0.08778**	**0.04**	**0.0665**	0.02	**0.0415**	0.02	**0.0324**	0.02
	Yearly	0.02654	0.06	.	.	.	.	.	.
Log Likelihood		-1251.6584		-260.5537		-324.2552		-273.8706	
LR χ^2		85.19		68.24		54.32		53.80	
Prob> χ^2		0.0003		0.0035		0.1370		0.1250	
Pseudo R^2		0.0329		0.1158		0.0773		0.0894	

[t]Bold font indicates significance with P-values ≤ 0.05.
[s]Marginal effects.
